# Statistical Reliability Analysis of Ultrasonic Velocity Method for Predicting Residual Strength of High-Strength Concrete under High-Temperature Conditions

**DOI:** 10.3390/ma17061406

**Published:** 2024-03-19

**Authors:** Wonchang Kim, Keesin Jeong, Taegyu Lee

**Affiliations:** Department of Fire and Disaster Prevention, Semyung University, Jecheon 27136, Choongbuk, Republic of Korea; kimwc69082@gmail.com

**Keywords:** concrete, ultrasonic pulse velocity, early age, high temperature, statistical analysis

## Abstract

Herein, we conducted a comprehensive statistical assessment of the ultrasonic pulse velocity (UPV) method’s effectiveness in predicting concrete strength under diverse conditions, specifically early age, middle age, and high-temperature exposure. The concrete mixtures, with water-to-cement (W/C) ratios of 0.33 and 0.28, were classified as granite aggregate or coal-ash aggregate mixes. Compressive strength and UPV measurements were performed under these conditions, and subsequent statistical analyses treated the identified factors as distinct groups. The results revealed a substantial difference in compressive strength between specimens at early age (average of 13.01) and those at middle age (average of 41.96) and after high-temperature exposure (average of 48.08). Conversely, UPV analysis showed an insignificant difference between the early-age specimens and those after high-temperature exposure. The analysis of the W/C ratio and coarse aggregate demonstrated significant differences (*p*-value < 0.05) in compressive strength between specimens in middle age and those exposed to high temperatures, excluding the early-age specimens. However, UPV analysis revealed insignificant differences, with *p*-values of 0.67 and 0.38 between specimens at an early age and post-high-temperature exposure, respectively. Regression analysis identified suitable functions for each scenario, emphasizing the importance of a strength prediction model for concrete after high-temperature exposure, particularly considering the W/C ratio. Since concrete showed statistically different compressive strength, UPV, and strength prediction models in three conditions (early age, middle age, and high temperature), different strength prediction models must be used for the purpose of accurately predicting the strength of concrete.

## 1. Introduction

Sound waves, characterized by mechanical pressure changes stemming from the elastic force, manifest through the periodic compression and restoration of particles within a medium via vibration. Sound waves with frequencies exceeding 20,000 Hz are termed “ultrasonic waves” [1]. These waves, which are beyond the audible range for humans, possess the ability to transmit signals imperceptible to the human ear. Inside the matrix and at the interface between concrete constituent materials, refraction, and reflection phenomena occur due to the influence of their short wavelengths. Therefore, measuring the velocity becomes feasible by examining the transmission time and distance traveled by the ultrasonic wave within the medium. This information is crucial for evaluating the quality of the targeted building members. The speed can be evaluated using Equation (1):

(1) $$v_{P}=\frac{L}{t}$$

where $v_{P}$ is velocity (m/s), L is distance (m), and t is time (s).

Given the potential drawbacks associated with traditional core extraction and destruction methods in concrete building evaluations in terms of evaluation time, equipment, manpower, cost, and required skill, engineers in the field are aiming for more economical concrete quality evaluation techniques using nondestructive testing, wherein the ultrasonic pulse velocity (UPV) technique has emerged as a popular approach among several researchers [2,3].

Table 1 provides a glimpse of previously proposed concrete strength prediction models utilizing UPV. These models can be broadly categorized into ‘early age’, ‘middle age’, and ‘high temperature’ [4,5,6,7,8,9,10,11,12,13,14,15,16]. Studies for predicting concrete strength using UPV, which were previously performed, can be divided into three main purposes. The early age model is used to evaluate the strength of concrete at an early age using UPV and to nondestructively gauge the time of vertical formwork demolding in the construction stage. To develop an early age model, researchers evaluated the compressive strength and UPV of concrete 24 h after concrete mixing and performed a regression analysis on the data, ultimately proposing a general exponential model [6,17].

The ‘middle age model’ measures the compressive strength and UPV after concrete mixing, and is derived through regression analysis of the collected data. Various types of models have been proposed using this approach, including linear and exponential. Notably, the ‘middle age model’ can be used to evaluate the quality of concrete in the maintenance and repair stages of a building, as well as to evaluate the demolding time of temporary materials in the construction stage.

Finally, a high-temperature model for predicting concrete strength after high-temperature exposure has been proposed based on UPV, leading to the derivation of various linear and power function models. To develop this model, researchers evaluated and performed regression analyses of the residual strength and UPV after concrete heating.

While various studies have attempted to formulate a comprehensive concrete strength prediction model using UPV, most have predominantly focused on comparing mechanical properties and model variances among different materials and mixing ratios. Unfortunately, there is a notable deficiency in analyses that explore the strength prediction model under diverse conditions (early age, middle age, and high temperature) and evaluate the discrepancies. UPV lacks a direct causal relationship with strength, but it is dominated by factors such as concrete voids, cracks, and elasticity of the matrix; thus, even with the same strength, different UPVs may appear depending on the matrix state [18,19,20].

Previous studies tested the statistical significance of the strength and UPV of heated and unheated concrete, and the results of the regression analysis were evaluated [21]. Concrete with a high-temperature history in the same UPV range showed a relatively high prediction rate in terms of compressive strength, and it was reported that crack formation owing to differences in the thermal expansion properties of mixed materials and voids owing to chemical decomposition contributed to the very low UPV [17,22]. Studies on the prediction of the strength of concrete at an early age using UPV have reported very low values, showing a different trend from the aforementioned research. Therefore, researchers and engineers must evaluate the relationship between concrete and UPV under various conditions to accurately predict the strength of concrete in different environments.

In this study, the strength and UPV of concrete under three conditions (early age, middle age, and high temperature) were evaluated The strength and UPV of concrete under three conditions (early age, middle age, and high temperature) were evaluated and the significance between the groups set up by statistically analyzing quantitative data was tested. The water-to-cement (W/C) ratio and coarse aggregate were mentioned as the most influential factors on the UPV of concrete, but the evaluation of the differences was somewhat insufficient quantitatively; therefore, they were also statistically reviewed in this study. Finally, a suitable model shape was derived through a regression analysis, and a concrete strength prediction model under each condition was proposed.

## 2. Experimental Plan and Methods

### 2.1. Experimental Plan

Table 2 presents the experimental design. The concrete types tested comprised concrete mixed with granite aggregate (GNA) and coal-ash aggregate (CAA). The target strength was set at 40 MPa (Korea’s high-strength concrete standard) and 8000 psi (55 MPa). The target strength adhered to both Korea’s high-strength concrete standard (40 MPa) and the American Concrete Institute (ACI) standard of 8000 psi (55 MPa). The W/C ratio was strategically set at 0.33 and 0.28 to meet the target strength requirements. The evaluation parameters included compressive strength and UPV. For the initial age assessment, the compressive strength and UPV of concrete were evaluated at 6, 12, 16, 20, and 24 h. Middle-age evaluations were conducted at 1, 7, 28, and 91 d, while assessments after high-temperature exposure spanned temperatures of 20, 200, 300, 500, and 700 °C, examining residual compressive strength and UPV. After evaluating the compressive strength and UPV, a statistical analysis was performed to examine the significance of the three conditions. The analysis tools used were Levene’s test, analysis of variance (ANOVA), post hoc test, regression analysis, and error test.

### 2.2. Materials

Table 3 summarizes the physical properties of the materials. As cement, ASTM Type-I ordinary Portland cement with a density of 3150 kg/m^3^ and a powder of 320 m^2^/kg was used. Granite coarse aggregates with a density of 2680 kg/m^3^, fineness of 7.03, and an absorption rate of 0.68% were used for GNA. For CAA, coal-ash artificial light aggregates with a density of 1470 kg/m^3^, fineness of 6.39, and an absorption rate of 8.68% were used.

**Figure 1:**
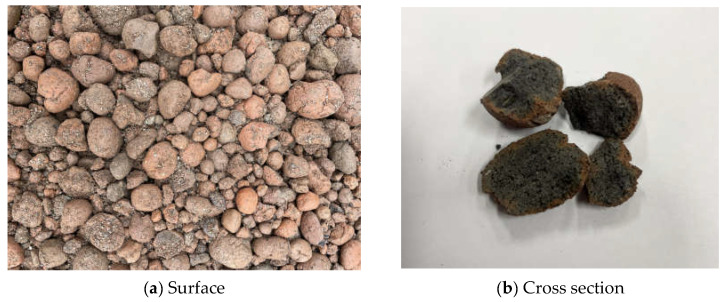
Coal-ash aggregate.

Figure 1 shows the lightweight coal-ash aggregate used in this study, which has a round surface and high porosity compared to commonly used crushed stone aggregates. River sand with a density of 2540 kg/m^3^, fineness of 2.54, and an absorption rate of 1.60% was used as the fine aggregate. A polycarboxylic-based superplasticizer was used to improve the workability of high-strength concrete. Table 4 lists the chemical composition of the cement used in this study.

### 2.3. Mix Proportion and Specimen Preparation

Table 5 summarizes the mix proportions used to prepare the cements in this study. Each specimen was named by combining the set concrete type (GNA and CAA) and W/C ratio (0.33, 0.28); the W/C ratio was set to 0.33 and 0.28 for target strength expression. In addition, to evaluate the effectiveness of the completely coarse aggregate type, the amounts of water, cement, and S/a were equal. The lightweight aggregate was pre-wetted in water for 24 h before mixing, and then dried at room temperature (20 ± 5 °C) for 12 h for a surface dry state of aggregate [23,24,25]. After mixing, cylinder concrete the specimen with Φ of 100 × 200 mm was produced to evaluate the mechanical properties of concrete under three conditions (early age, middle age, and high temperature) [26]. After manufacture, the specimen was cured in water until 28 d of age, and then cured in a chamber of room temperature (20 ± 5 °C) and humility (50 ± 5%) until 91 d of age. Before evaluating the compressive strength and UPV of the specimens, both ends of the concrete were polished with an abrasive to prevent eccentricity.

### 2.4. Test Method

**Figure 2:**
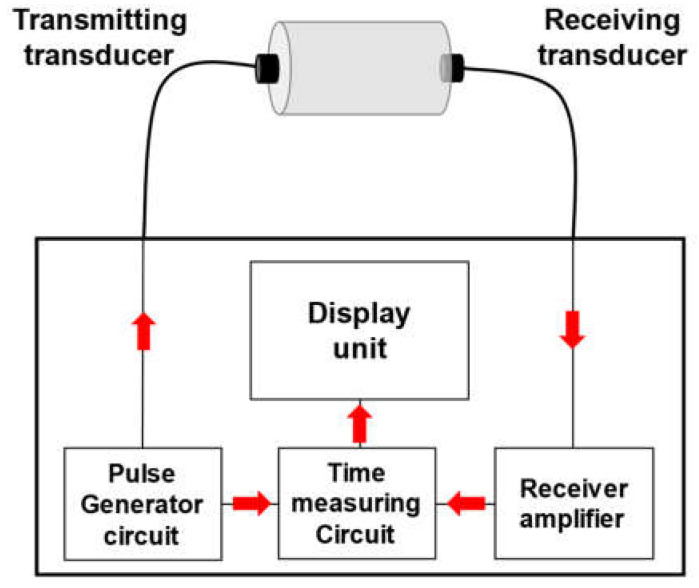
Procedure of ultrasonic pulse velocity test [29].

The compressive strength of the specimens was evaluated based on ASTM C39/C39M, and the UPV was evaluated based on ASTM C597-16 [27,28]. Figure 2 shows the UPV evaluation method using an Ultrasonic Concrete Tester Ultracon-170 manufactured by M.K.C KOREA (Seoul, Republic of Korea) [29]. A grease was applied for close contact between both ends of the specimen and the transducer. The results of the mechanical properties are the average of three specimens.

An electric furnace was used for heating the specimen curing for 91 days, and the heating rate was set to 1 °C/min and heated to the target temperature (200, 300, 500, and 700 °C). After reaching the target temperature, it was maintained at the target temperature for 60 min to ensure a uniform temperature distribution in the specimen. After that, the cover of the electrical furnace was opened and cooled at low speed in the air until the temperature of the specimen reached 20 ± 5 °C, and the mechanical properties were evaluated [30].

To evaluate the statistical significance of the compressive strength and UPV of concrete under the three conditions, each was first set and classified according to the type of concrete, W/C ratio, and three conditions [31]. Subsequently, Levene’s test, a method of testing equal variance for three or more groups, was performed on each group, and significant differences between groups were evaluated by performing “ANOVA” and “Welch’s ANOVA” according to the test results. ANOVA was used to test the significance between groups through the average of three or more groups when equal variance was shown according to the results of Levene’s test, and Welch’s ANOVA was performed when heteroscedasticity was shown. After performing the ANOVA, a post hoc test was used to evaluate which of the three or more groups showed significant differences. After evaluating the significance between the groups, regression analysis and error tests for compressive strength and UPV were performed. After approximately 12 h, the specimen with a W/C ratio of 0.28 showed lower strength than the specimen with a W/C ratio of 0.33, owing to the effect of the setting delay of the admixture to improve workability.

## 3. Results and Discussion

### 3.1. Mechanical Properties of Concrete

#### 3.1.1. Compressive Strength

**Figure 3:**
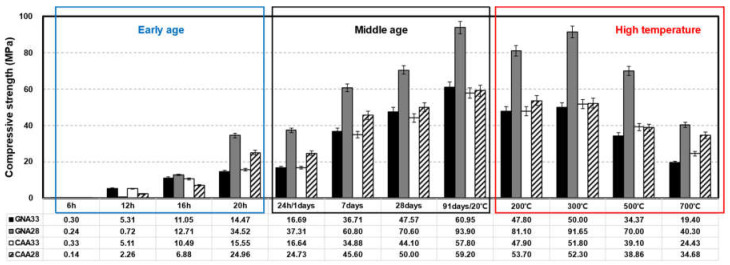
Result of compressive strength on concrete.

Figure 3 shows the results for the concrete compressive strength under the three conditions. The compressive strength converged to zero because none of the specimens that elapsed for approximately 6 h at an early age expressed their properties as elastic bodies [32]. After approximately 16 h, all specimens except CAA28 expressed approximately 10 MPa. After approximately 20 h, GNA28 showed about 20.05 MPa higher than that of GNA33, and CAA28 showed about 9.41 MPa higher strength than CAA33.

Throughout the curing period from day 1 to 91, GNA28 exhibited an approximately 72.93% higher intensity expression compared to GNA33, while CAA28 showed an approximately 23.79% higher intensity expression than CAA33. Although GNA33 and CAA33 showed similar strength expressions in the early and middle ages, the difference in compressive strength between GNA28 and CAA28 increased with age. In the case of coal-ash artificial lightweight aggregates mixed with CAA, aggregates are developed by the molding and calcination processes of bottom ash and dredging soil generated under the boilers of thermal power plants, contributing to the low strength of concrete compared to normal aggregates owing to the influence of porosity [33]. When load loading was applied to CAA28, the lightweight aggregate broke before the mortar; therefore, it showed a very low compressive strength compared to GNA28 mixed with relatively strong aggregates [34]. In addition, because there was only a difference of approximately 1.40 MPa between CAA33 and CAA28, when cement was used as a binder up to a certain W/C ratio, the strength of the lightweight aggregate concrete would be compromised owing to the material limitations of lightweight aggregates.

**Figure 4:**
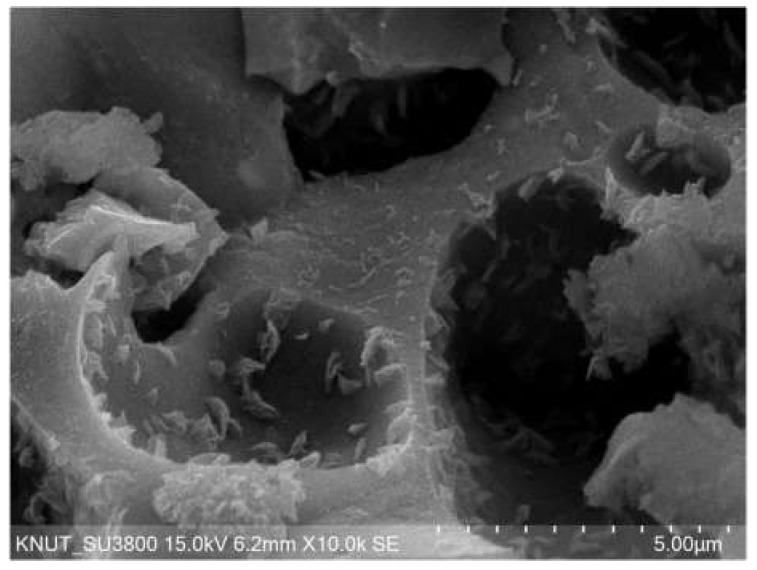
SEM result of coal-ash aggregate.

During the initial stage up to approximately 16 h, both GNA and CAA exhibited a lower tendency concerning the difference in compressive strength compared to the middle age. Specifically, GNA33 and CAA33 demonstrated similar strengths, maintaining this parity even as time progressed. This divergence in behavior prompted an investigation into the properties of these aggregates. In previous studies, cement particles penetrated the voids in the lightweight aggregate during the curing process and the hydration process inside the lightweight aggregate, and high adhesion of mortar and aggregate was reported owing to this effect [35,36]. Figure 4 shows the SEM results of the coal-ash aggregate. A hydration product crystal phase generated during the cement hydration process was found inside the pores of the aggregate. However, in the case of concrete mixed with normal aggregate, the wall effect, which is a crack at the interface between the mortar and aggregate, has been reported [37,38]. Owing to the properties of these two aggregates, it was determined that the difference in strength between ordinary concrete and lightweight concrete was small at an early age and for low-target-strength concrete.

After high-temperature exposure, the specimen showed a strength decrease of approximately 15.41% up to 200 °C; conversely, a strength increase of ~5.79% was observed at 300 °C. A similar trend was reported in previous studies. Lee et al. reported the rehydration of unreacted products due to high temperature and high pressure, coupled with the complex action of thermal expansion stress between mixed materials [39,40]. At temperatures above 300 °C, the strength continued to decrease, with CAA33 exhibiting a compressive strength approximately 57.19% higher than that of GNA33. GNA28 showed high residual strength in all temperature ranges compared to CAA28; however, CAA28 and GNA28 showed residual strength rates of approximately 75.82% and 70.60%, respectively. Using SEM analysis, Roufael et al. reported that cracks at the interface between coarse aggregates and mortar were improved by the influence of small thermal expansion properties, owing to the porosity of lightweight aggregates. Lee et al. reported that lightweight concrete subjected to high temperatures exhibited higher residual strength and smaller thermal expansion deformation than regular concrete [39,41].

#### 3.1.2. Ultrasonic Pulse Velocity

**Figure 5:**
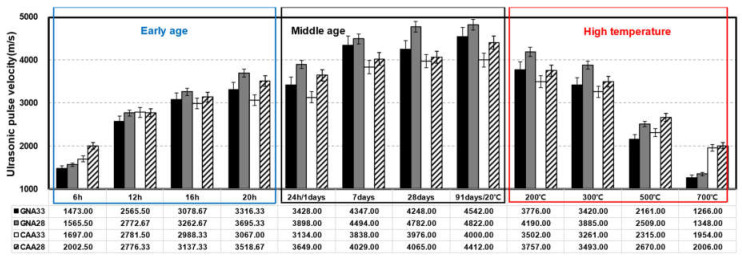
Result of ultrasonic pulse velocity on concrete.

Figure 5 shows the UPV results for concrete under the conditions of early age, middle age, and high-temperature exposure. The UPV tended to be higher under a low W/C ratio and high elasticity of the mixed coarse aggregate.

From approximately 16 h into the early age stage, a consistent trend emerged where specimens with a W/C ratio of 0.28 exhibited higher UPV compared to those with a W/C ratio of 0.33. Additionally, within the same W/C ratio, GNA consistently displayed higher UPV values compared to CAA. This trend persisted into middle age, and by 91 d of aging, specimens with a W/C ratio of 0.28 demonstrated roughly 8.72% higher UPV than those with a W/C ratio of 0.33. Similarly, under the same W/C ratio, GNA showed approximately 11.04% higher UPV than CAA. The sensitivity of UPV to voids and cracks within the concrete matrix was evident, with lower W/C ratios leading to higher UPV due to the influence of a relatively dense matrix. Moreover, the presence of a relatively porous lightweight aggregate in CAA resulted in lower UPV compared to GNA (refer to Figure 4) [42,43].

After high-temperature exposure, UPV continued to decrease with the increasing temperature, with CAA showing a higher residual UPV than GNA at the same W/C ratio in the temperature range beyond ~500 °C. At approximately 700 °C, CAA showed a UPV that was ~4.57% higher than that of GNA, indicating a potential mitigation of crack generation compared to GNA due to the smaller thermal expansion deformation of lightweight aggregates. In addition, CAA exhibited a residual rate approximately 10.35% higher than that of GNA as the temperature increased [38,41].

### 3.2. Statistical Analysis

#### 3.2.1. Statistical Significance Test for 3 Conditions

In this phase of the study, statistical analysis methods were employed to evaluate the significance of three critical conditions: W/C ratio and density of coarse aggregates. Each of these factors was treated as an individual group. First, Levene’s test, an equal variance evaluation method of three or more groups, was performed. A ‘*p* value of <0.05′ implied heteroscedasticity between groups, while a value of 0.05 or more suggested equal variance. Based on the results of Levene’s test, ANOVA for equal variance and Welch’s ANOVA for heteroscedasticity were performed. ANOVA (Welch’s ANOVA) was used to evaluate the significance of three or more groups through the corresponding degree of variance. A significant difference between groups was considered for *p*-values < 0.05, while no significant difference was considered for *p*-values below 0.05. Therefore, in the process of statistically testing the null hypothesis and the alternative hypothesis (experimental hypothesis), the null hypothesis was set as showing no significant difference in compressive strength and UPV by each set factor (three conditions, W/C ratio, and thick aggregate density), and the experimental hypothesis was set to showing a significant difference by each set factor.

**Figure 6:**
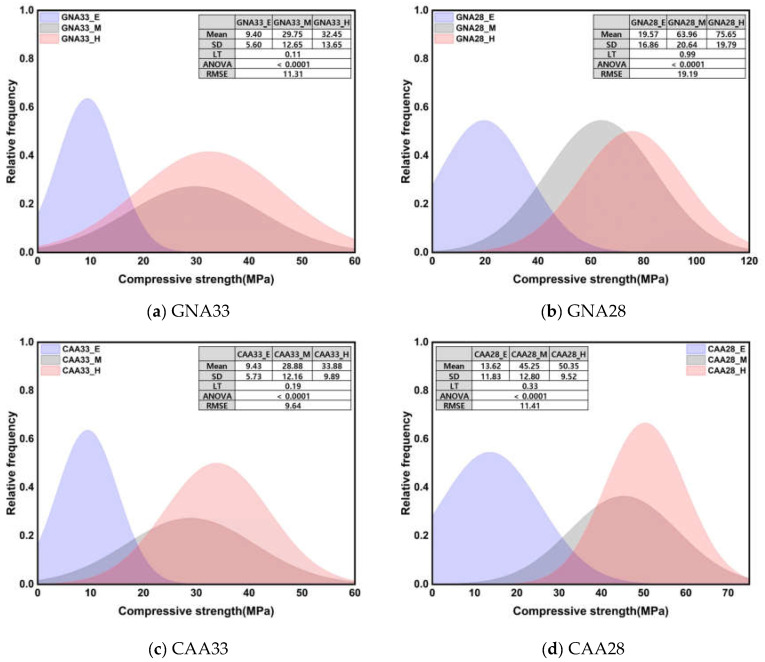
Normal distribution of compressive strength and results of significance test according to 3 conditions.

Figure 6 shows the normal distribution of compressive strength and the results of the significance test according to three conditions. The results of the compressive strength analysis under the three conditions are shown as mean, standard deviation, Levene’s test results, ANOVA, and root mean square error (RMSE). All Leven’s test results showed equal variance with a *p*-value of 0.05 or more for all specimens, and the resulting ANOVA results showed a significant difference between groups with a *p*-value of 0.0001 or less. The compressive strength at an early age showed a notably low mean compared to other conditions, while the mean at middle age and after high-temperature treatment was comparable. The standard deviation also showed the lowest value at an early age, stemming from the influence of the intensity expression range. Notably, GNA28 displayed the highest standard deviation value, attributable to the wider intensity expression range compared with the other specimens. Additionally, the RMSE results highlighted that GNA28 had the highest value among the groups.

**Figure 7:**
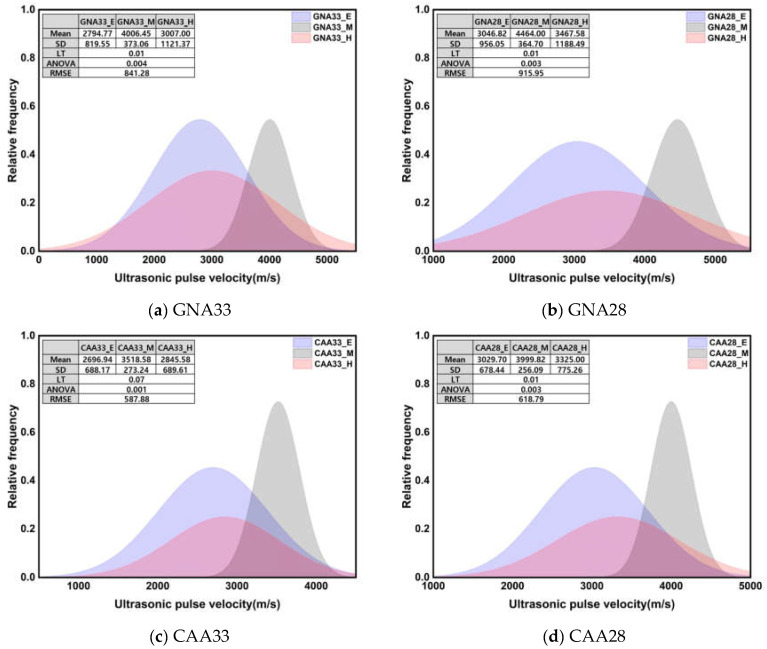
Normal distribution of UPV and results of significance test according to 3 conditions.

Figure 7 shows the normal distribution of UPV and the results of the significance test according to three conditions. All Levene’s test results showed heteroscedasticity with a *p*-value of 0.05 or less, and Welch’s ANOVA results also showed a significant difference between groups with a *p*-value of 0.05 or less. Unlike the compressive strength, the average and standard deviation of the UPV showed similar values at an early age and after high-temperature treatment. Under the early age and high-temperature conditions, the UPV mean was approximately 24.11% lower than that under the middle-age condition, while the standard deviation was approximately 273% higher. The observed disparities in the statistical results can be attributed to the distinct behavior of UPV at an early age compared to compressive strength: at an early age, UPV exhibited a relatively high rate of increase over the course of the curing time, unlike compressive strength. Meanwhile, during high-temperature treatment, UPV decreased significantly, indicating severe deterioration. The impact of high temperature on UPV, in contrast to compressive strength, is particularly noteworthy and contributes to the observed variations in the statistical outcomes.

ANOVA was used to test the significance of more than three groups, but a post hoc test was necessary to test which groups had significant differences. Table 6 presents the post hoc test results for Figure 6 and Figure 7. The compressive strength showed significant differences for all specimens in the middle age and high-temperature conditions, while the UPV showed significant differences in the early age and high-temperature conditions. Thus, the compression strength and UPV factors under the three conditions were considered to have an important influence on the regression analysis results owing to the statistically different results between groups.

#### 3.2.2. Statistical Significance Test according to W/C Ratio and Coarse Aggregate

**Figure 8:**
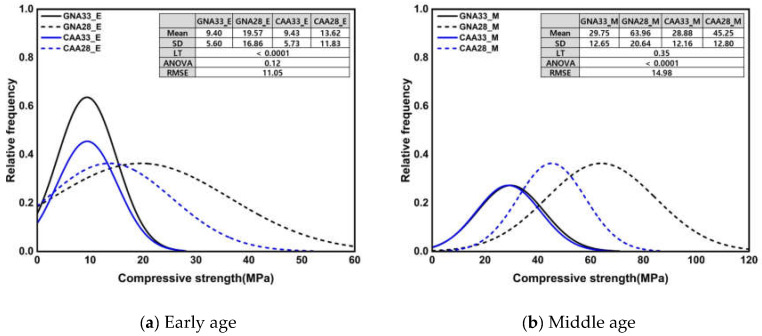

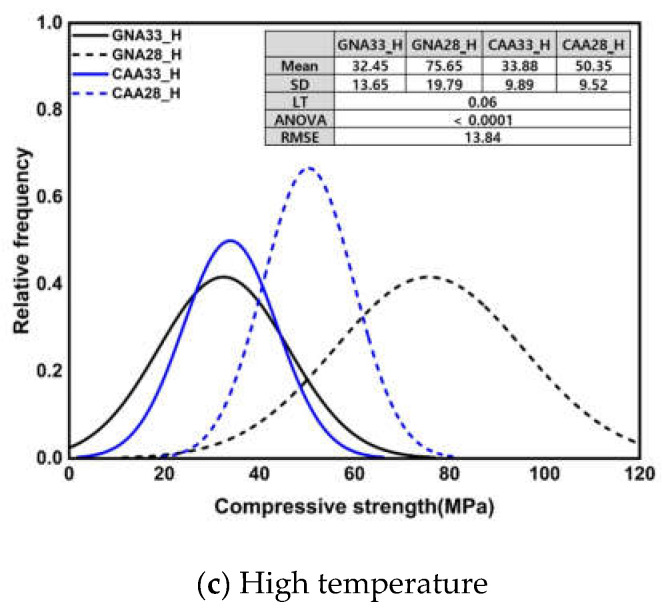
Normal distribution of compressive strength and results of significance test according to W/C ratio and coarse aggregate.

Figure 8 shows the normal distribution of the compressive strength and the results of the significance test according to the three conditions. Under all conditions, the mean and standard deviation of GNA33 and CAA33 were similar; however, compared to CAA28, GNA28 showed a markedly higher mean of approximately 45.08% and a higher standard deviation of approximately 70.56%. In addition, the mean and standard deviation of the specimen with a W/C ratio of 0.28 were higher by approximately 84.31% and 69.51%, respectively, compared to those of the specimen with a W/C ratio of 0.33. Only the results of Levene’s test on compressive strength at an early age suggested heteroscedasticity behavior under a *p*-value of 0.05 or less; nevertheless, the ANOVA results showed insignificant results with a *p*-value of 0.05 or more. Additionally, the RMSE values were similar under all conditions.

**Figure 9:**
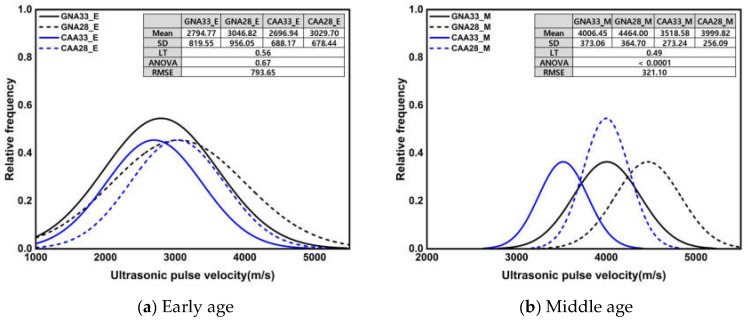

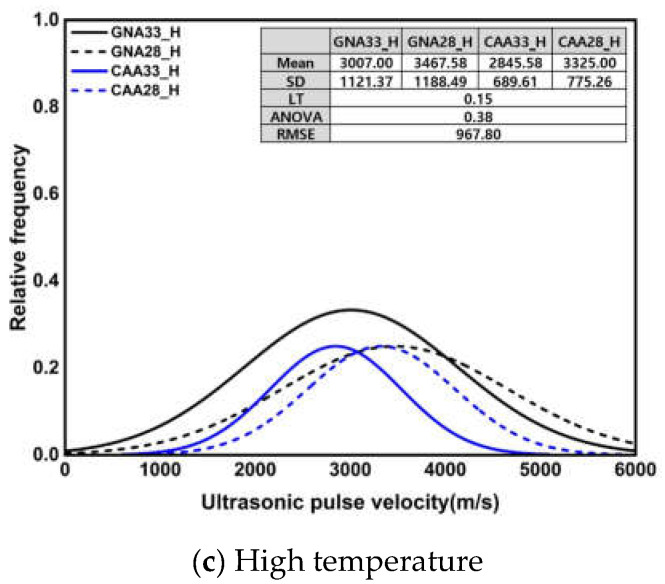
Normal distribution of UPV and results of significance test according to W/C ratio and coarse aggregate.

Table 7 presents the post hoc test results for Figure 8 and Figure 9. As can be seen, there were no significant differences in the compressive strength and UPV values at an early age. The compressive strength in middle age showed insignificant results, except for GNA28, and the UPV showed significant differences between the groups, except for GNA33 and CAA28. The compressive strength of the specimens after high-temperature treatment showed a significant difference between the groups, except for GNA33 and CAA33, but UPV did not show a significant difference in any of the specimens. The statistical analysis results for the W/C ratio and coarse aggregates showed different significances between the groups in terms of compressive strength and UPV. This suggested that even under the same compressive strength, different UPV values may occur, and, on the contrary, different compressive strengths may appear under the same UPV. Therefore, it is necessary to consider these factors in the strength prediction model through regression analysis.

#### 3.2.3. Regression Analysis Results

Table 8 presents the regression analysis results using the iteration algorithm of Levenberg Marquardt provided by OriginPro 2023. The *p*-value of both GNA and CAA in the early age was 0.05 or less; meanwhile, the R-square (R^2^) value was higher in the exponential function model, and the RMSE value was higher in the linear function model. Most previous studies that proposed a strength prediction model using the UPV at an early age also suggested an exponential function model. The strength prediction models for GNA and CAA in middle-aged specimens exhibited different forms. Particularly, the GNA model favored an exponential function, whereas the CAA model found suitability in the linear function. After exposure to high temperatures, both the GNA and CAA models showed suitable linear functional forms, albeit with lower R^2^ and higher RMSE values than those in other models. In the case of post-high-temperature exposure, there was a significant difference in the compressive strength according to the W/C ratio; however, UPV followed the opposite trend. This implied that concrete with a low W/C ratio under a constant UPV exhibits a high compressive strength, which agrees well with the results from previous studies [21]. Therefore, the W/C ratio is highlighted as an important factor in the development of a concrete strength prediction model using UPV after high-temperature exposure [44,45,46,47].

**Figure 10:**
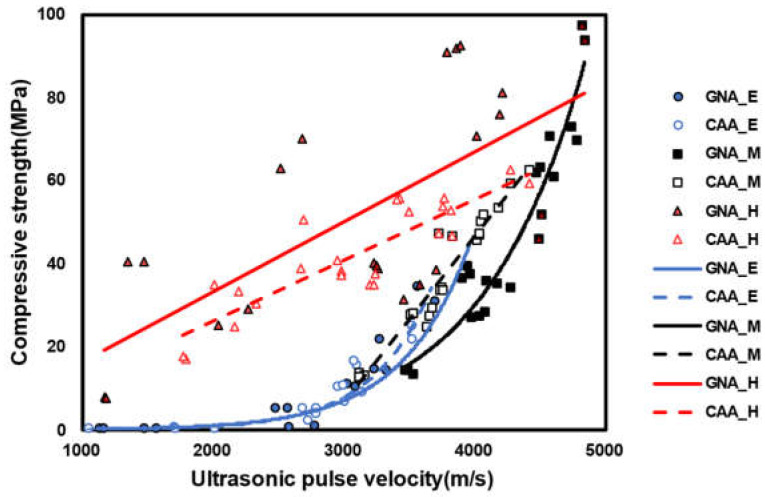
Regression model of concrete.

Figure 10 illustrates the regression model of concrete, and the parameters for each model are summarized in Table 9. The regression models of GNA and CAA after high-temperature exposure confirmed very large data deviations.

**Figure 11:**
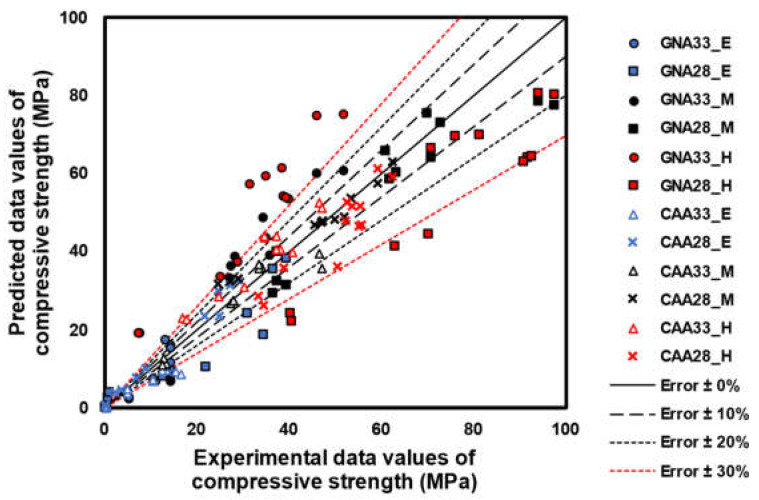
Error test.

Figure 11 shows the error test results for the model shown in Figure 10. This was tested by setting the experimental data on the x-axis and the prediction data derived from the model on the y-axis. At an early age, the CAA specimen showed a small error compared with its GNA equivalent because the W/C ratio was statistically insignificant. However, because GNA33 and GNA28 showed statistically significant differences in terms of the W/C ratio, GNA33 showed high prediction accuracy, in contrast to GNA28.

Conversely, CAA predominantly yielded insignificant results for the W/C ratio factor, owing to the limitation of the strength development of concrete due to the stiffness of the aggregate. The error deviation compared to that of GNA was found to be small. Despite previous research on the strength prediction of concrete using UPV, no clear conclusion has been drawn regarding the conditions under which the strength prediction will be performed on concrete. In addition, because the quantitative analysis of the W/C ratio or mixed material is insufficient, determining whether a model considering the W/C ratio or mixed material needs to be proposed is inferred to be a challenging task. Therefore, a statistically large number of factors must be continuously analyzed to accurately predict the strength of concrete for this purpose.

## 4. Conclusions

In this study, the compressive strength and UPV of concrete under three conditions (early age, middle age, and high-temperature exposure) were measured and statistically analyzed, and the significance between the set factors was tested. More importantly, a model for predicting the strength of concrete using UPV was proposed through regression analysis, with the following conclusions drawn:

(1) During the early age of 16 h, all specimens exhibited similar compressive strengths. However, from 1 to 91 d of age, GNA28 showed approximately 72.93% higher strength than GNA33, and CAA28 showed approximately 23.79% higher strength than CAA33. After high-temperature exposure, the lightweight aggregate exhibited a higher residual rate than normal concrete, owing to the influence of small thermal expansion deformation.
(2) At approximately 16 h, at an early age, the specimen with a W/C ratio of 0.28 showed a higher UPV than the specimen with a W/C ratio of 0.33; by 91 d of age, GNA showed approximately 8.72% higher UPV owing to the influence of the porous lightweight aggregate mixed with CAA. After high-temperature exposure, CAA showed a residual rate approximately 10.35% higher than that of GNA.
(3) The compressive strength of all specimens showed a very low mean at an early age, with no significant difference between strength at middle age, and that after high-temperature exposure. UPV showed the highest mean at middle age among the three conditions, while also showing a similar average at early age and after high-temperature exposure; therefore, it was inferred as a statistically identical group.
(4) In the ANOVA result of the compressive strength at an early age, the *p*-value was 0.05 or more, indicating insignificance. However, in middle age, all compressive strengths were significant except for GNA28. The compressive strengths of the specimens after high-temperature exposure exhibited significant differences between the groups, except for GNA33 and CAA33. On the contrary, UPV did not show a significant difference for concrete at an early age or after high-temperature exposure, except for the middle-age condition.
(5) Regression analyses revealed that the exponential function forms were suitable for GNA and CAA at the early stage, and GNA at middle age. On the contrary, the linear functional forms showed optimal suitability for CAA and GNA after high-temperature exposure, and CAA at middle age. However, after high-temperature exposure, the GNA and CAA groups showed very low R-square values, primarily attributed to their dependence on the W/C ratio; as such, it is generally acknowledged that the W/C ratio of lightweight concrete assumes higher importance in model consideration.

In this study, the significance of the concrete strength prediction models in ‘early age’, ‘middle age’, and ‘high temperature’ was evaluated only statistically. In order to evaluate this from a material point of view, it is necessary to perform a qualitative evaluation of the matrix structure and deterioration state by performing ‘SEM’ and ‘porosimetry’ analysis. In addition, qualitative and quantitative evaluation of cement hydrates and other chemical compositions such as XRD and XRF is required. Currently, researchers using UPV to conduct research on concrete strength prediction are proposing a strength prediction model using machine learning-based computer analysis, and future studies will conduct a study that combines micro-analysis and AI analysis techniques of concrete.

In addition, research on cement alternative materials for greenhouse gas reduction has been actively conducted recently, and research on strength prediction models for concrete mixed with various mixtures (fly ash, blast furnace slag, clay, etc.) will also be conducted.

## Figures and Tables

**Table 1:** Previous prediction model of concrete strength based on UPV.

Type	Researchers	Equations of Model
Early age	R. L. Al-Mufti	$f_{c}=0.0063\times e^{1.8796\times UPV}\;f_{c}=0.0125\times e^{1.6754\times UPV}\;f_{c}=0.0098e^{1.7456\times UPV}$
	I. Lawson	$f_{c}=0.022\times e^{0.001\times UPV}\;f_{c}=0.053\times e^{0.001\times UPV}\;f_{c}=0.097\times e^{0.001\times UPV}\;f_{c}=0.205\times e^{0.001\times UPV}$
	T. Lee	$f_{c}=1.25\times e^{0.04\times UPV}$
Middle age	R. K. Majhi	$f_{c}=12.137\times UPV-21.619$
	P. Shafigh	$f_{c}=0.072\times e^{1.543\times UPV}$
	G. Sua-iam	$f_{c}=18.311\times UPV-29.114$
	S. Kou	$f_{c}=0.2158\times e^{0.0012\times UPV}$
High temperature	N. V. S. Kumar	$f_{c}=6.6502\times UPV-0.2142$
	A. K. Saha	$f_{c}=11.5\times {UPV}^{1.2}$
	M. Z. Islam	$f_{c}=12.616\times UPV-12.48$
	U. Dolinar	$f_{c}=15.06\times {UPV}^{1.195}$

**Table 2:** Experimental plan outline.

Item	Details
Types of concrete	GNA (Granite aggregate) CAA (Coal-ash aggregate)
Water–cement ratio	0.33, 0.28
Elapsed time at early age	6, 12, 16, 20, 24 h
Curing age	1, 7, 28, 91 d
Heating temperature	20, 200, 300, 500, 700 °C
Mechanical properties	Compressive strength UPV (Ultrasonic pulse velocity)
Statistical analysis	Levene’s test, ANOVA (or Welch’s ANOVA), Post hoc test, Regression analysis, Error test

**Table 3:** Physical properties of materials.

Item	Details
Cement	ASTM Type-I ordinary Portland cement Density: 3150 kg/m^3^, fineness: 320 m^2^/kg
Coarse aggregate	Granite aggregate Density: 2680 kg/m^3^, fineness modulus: 7.03, absorption: 0.68%, Size_max_: 20 mm
	Coal-ash aggregate Density: 1470 kg/m^3^, fineness modulus: 6.39, absorption: 8.68%, Size_max_: 20 mm
Fine aggregate	River sand Density: 2540 kg/m^3^, fineness modulus: 2.54, absorption: 1.60%
Super plasticizer (SP)	Polycarboxylic-based super plasticizer

**Table 4:** Chemical composition of cement.

Materials	Chemical Compositions (%)								
	CaO	SiO_2_	Al_2_O_3_	Fe_2_O_3_	MgO	SO_3_	K_2_O	Others	L.O.I
OPC ^(1)^	60.30	19.80	4.90	3.30	3.80	2.90	1.10	0.90	3.00

^(1)^ OPC: ordinary Portland cement.

**Table 5:** Mix proportions of concrete.

Mix ID	Ratio		Unit Weight (kg/m^3^)				
	W/C ^(1)^	S/a ^(2)^	Water	Cement	River Sand	Granite Aggregate	Coal-Ash Aggregate
GNA33	0.33	0.43	165	500	711	762	-
GNA28	0.28			500	711	-	533
CAA33	0.33			600	676	896	-
CAA28	0.28			600	676	-	507

^(1)^ W/C (Water/Cement); ^(2)^ S/a (Sand/aggregate).

**Table 6:** Post hoc test for Figure 6 and Figure 7.

ID	Mean Difference		*p*-Value		ID	Grouping Letters Table	
	Comp.	UPV	Comp.	UPV		Comp.	UPV
GNA33_E & GNA33_M	20.35	1211.68	0.001	0.006	GNA33_E	A	A
GNA33_E & GNA33_H	23.06	212.23	<0.001	1.000	GNA33_M	A	B
GNA33_M & GNA33_H	2.70	999.45	1.000	0.023	GNA33_H	B	B
GNA28_E & GNA28_M	44.39	1417.18	<0.001	0.003	GNA28_E	A	A
GNA28_E & GNA28_H	56.08	420.77	<0.001	0.84	GNA28_M	A	B
GNA28_M & GNA28_H	11.69	996.42	0.46	0.04	GNA28_H	B	B
CAA33_E & CAA33_M	19.45	821.64	<0.001	0.007	CAA33_E	A	A
CAA33_E & CAA33_H	24.45	148.64	<0.001	0.818	CAA33_M	A	B
CAA33_M & CAA33_H	5.00	672.99	0.67	0.026	CAA33_H	B	B
CAA28_E & CAA28_M	31.63	970.12	<0.001	0.003	CAA28_E	A	A
CAA28_E & CAA28_H	36.73	295.30	<0.001	0.785	CAA28_M	A	B
CAA28_M & CAA28_H	5.10	674.82	0.539	0.041	CAA28_H	B	B

**Table 7:** Post hoc test for Figure 8 and Figure 9.

ID	Mean Difference		*p*-Value		ID	Grouping Letters Table	
	Comp.	UPV	Comp.	UPV		Comp.	UPV
GNA33_E & GNA28_E	10.17	252.05	0.221	1.000	GNA33_E	A	A
GNA33_E & CAA33_E	0.04	97.86	1.000	1.000	GNA28_E	A	A
GNA28_E & CAA33_E	10.13	349.88	0.226	1.000	CAA33_E	A	A
GNA33_E & CAA28_E	4.22	234.92	1.000	1.000	CAA28_E	A	A
GNA28_E & CAA28_E	5.95	17.12	1.000	1.000	-		
CAA33_E & CAA28_E	4.19	332.76	1.000	1.000	-		
GNA33_M & GNA28_M	34.20	457.55	<0.001	0.011	GNA33_M	B	B
GNA33_M & CAA33_M	0.87	487.88	1.000	0.006	GNA28_M	A	A
GNA28_M & CAA33_M	35.07	945.42	<0.001	<0.001	CAA33_M	B	C
GNA33_M & CAA28_M	15.50	6.64	0.119	1.000	CAA28_M	B	B
GNA28_M & CAA28_M	18.70	464.18	0.034	0.009	-		
CAA33_M & CAA28_M	16.37	481.24	0.086	0.007	-		
GNA33_H & GNA28_H	43.19	460.58	<0.001	1.000	GNA33_H	C	A
GNA33_H & CAA33_H	1.43	161.42	1.000	1.000	GNA28_H	A	A
GNA28_H & CAA33_H	41.76	622.00	<0.001	0.736	CAA33_H	C	A
GNA33_H & CAA28_H	17.89	318.00	0.017	1.000	CAA28_H	B	A
GNA28_H & CAA28_H	25.30	142.58	<0.001	1.000	-		
CAA33_H & CAA28_H	16.46	479.42	0.034	1.000	-		

**Table 8:** Results of regression analysis.

ID	Model	*p*-Value	Pearson’s r	R-Square	RMSE
GNA_E	Linear	<0.0010	0.82	0.67	7.89
	Exponential	<0.0001	-	0.89	4.52
GNA_M	Linear	<0.0001	0.92	0.85	9.49
	Exponential	<0.0001	-	0.91	7.36
GNA_H	Linear	0.0001	0.70	0.49	20.09
	Exponential	1.0000	-	−3.99	63.13
CAA_E	Linear	<0.0001	0.84	0.71	5.16
	Exponential	<0.0001	-	0.93	2.50
CAA_M	Linear	<0.0001	0.96	0.93	4.03
	Exponential	<0.0001	-	0.90	4.85
CAA_H	Linear	<0.0001	0.88	0.77	6.26
	Exponential	1.0000	-	−11.51	45.86

**Table 9:** Equation and coefficient of the regression model in Figure 10.

ID		Types of Models	Equation	Regression Coefficient (R^2^)
Early age	GNA	Exponential function	$y=0.0218\times e^{0.0019\times UPV}$	0.89
	CAA		$y=0.0103\times e^{0.0022\times UPV}$	0.93
Middle age	GNA		$y=0.1752\times e^{0.0013\times UPV}$	0.91
	CAA	Linear function	y=0.0399×UPV−113.02	0.93
High temperature	GNA		y=0.0168×UPV−0.4195	0.49
	CAA		y=0.0146×UPV−3.0737	0.77

## Data Availability

The data presented in this study are available on request to the corresponding author.

## References

[1] Baehaki, Andi M., Yohanes G.R. (2019). Experimental study of crack depth measurement of concrete with ultrasonic pulse velocity (UPV). IOP Conf. Ser. Mater. Sci. Eng..

[2] Kim S.D., Shin D.H., Lim L.M., Lee J., Kim S.H. (2005). Designed Strength Identification of Concrete by Ultrasonic Signal Processing Based on Artificial Intelligence Techniques. IEEE Trans. Ultrason. Ferroelectr. Freq. Control..

[3] Bonamy D., Bounchaud E. (2011). Failure of heterogeneous materials: A dynamic phase transition?. Phys. Rep..

[4] Al-Mufti R.L., Fried A.N. (2018). Non-destructive evaluation of reclaimed asphalt cement concrete. Eur. J. Environ. Civ. Eng..

[5] Lawson I., Danso K.A., Odoi H.C., Adjei C.A., Quashie F.K. (2011). Non-Destructive Evaluation of Concrete using Ultrasonic Pulse Velocity. Res. J. Appl. Sci. Eng. Technol..

[6] Lee T., Lee J. (2020). Setting time and compressive strength prediction model of concrete by nondestructive ultrasonic pulse velocity testing at early age. Constr. Build. Mater..

[7] Majhi R.K., Padhy A., Nayak A.N. (2021). Performance of structural lightweight concrete produced by utilizing high volume of fly ash cenosphere and sintered fly ash aggregate with silica fume. Clean. Eng. Technol..

[8] Shafigh P., Nomeli M.A., Alengaram U.J., Mahmud H.B., Jumaat M.Z. (2016). Engineering properties of lightweight aggregate concrete containing limestone powder and high volume fly ash. J. Clean. Prod..

[9] Sua-iam G., Sokrai P., Makul N. (2016). Novel ternary blends of Type 1 Portland cement, residual rice husk ash, and limestone powder to improve the properties of self-compacting concrete. Constr. Build. Mater..

[10] Kou S.C., Poon C.S., Wan H.W. (2012). Properties of concrete prepared with low-grade recycled aggregates. Constr. Build. Mater..

[11] Kumar V.S., Ram K.S.S. (2019). Performance of Concrete At Elevated Temperatures Made With Crushed Rock Dust As Filler Material. Mater. Today Proc..

[12] Saha A.K., Sarker P.K., Majhi S. (2019). Effect of elevated temperatures on concrete incorporating ferronickel slag as fine aggregate. Fire Mater..

[13] Islam M.Z., Sohel K.M.A., Al-Jabri K., Harthy A.A. (2021). Properties of concrete with ferrochrome slag as a fine aggregate at elevated temperatures. Case Stud. Constr. Mater..

[14] Dolinar U., Trtnik G., Turk G., Hozjan T. (2019). The feasibility of estimation of mechanical properties of limestone concrete after fire using nondestructive methods. Constr. Build. Mater..

[15] Sevim O., Alakara E.H., Demir I., Bayer I.R. (2023). Effect of magnetic water on properties of slag-based geopolymer composites incorporating ceramic tile waste from construction and demolition waste. Arch. Civ. Mech. Eng..

[16] Sevim O., Alakara E.H., Guzelkucuk S. (2023). Fresh and Hardened Properties of Cementitious Composites Incorporating Firebrick Powder from Construction and Demolition Waste. Buildings.

[17] Nam Y., Jeong K., Kim W., Choi H., Lee T. (2023). Evaluation on Early Strength Development of Concrete Mixed with Non-Sintered Hwangto Using Ultrasonic Pulse Velocity. Materials.

[18] Zhang Y., Aslani F. (2021). Compressive strength prediction models of lightweight aggregate concretes using ultrasonic pulse velocity. Constr. Build. Mater..

[19] Khan K., Amin M.N., Sahar U.U., Ahmad W., Shah K., Mohamed A. (2022). Machine learning techniques to evaluate the ultrasonic pulse velocity of hybrid fiber-reinforced concrete modified with nano-silica. Front. Mater..

[20] Tenza-Abril A.J., Villacampa Y., Solak A.B., Baeza-Brotons F. (2018). Prediction and sensitivity analysis of compressive strength in segregated lightweight concrete based on artificial neural network using ultrasonic pulse velocity. Constr. Build. Mater..

[21] Kim W., Lee T. (2023). A Study to Improve the Reliability of High-Strength Concrete Strength Evaluation Using an Ultrasonic Velocity Method. Materials.

[22] Lee H.K., Lee K.M., Kim Y.H., Yim H., Bae D.B. (2004). Ultrasonic in-situ monitoring of setting process of high-performance concrete. Cem. Concr. Res..

[23] Ji G.B., Mun J.H., Yang K.H. (2019). Evaluation of Mechanical Properties of Lightweight Concrete Using Bottom Ash Aggregates and Foam. J. Korea Concr. Inst..

[24] Lee K.H., Yang K.H. (2018). Proposal for Compressive Strength Development Model of Lightweight Aggregate Concrete Using Expanded Bottom Ash and Dredged Soil Granules. Archit. Inst. Korea.

[25] Choi S.J., Kim D.B., Lee K.S., Kim Y.U. (2019). The Study on the Physical and Strength Properties of Lightweight Concrete by Replacement Ratio of Artificial Lightweight Aggregate. J. Korea Inst. Build. Constr..

[26] (2015). Standard Test Method for Compressive Strength of Concrete Cylinders Cast in Place in Cylindrical Molds.

[27] (2018). Standard Test Method for Compressive Strength of Cylindrical Concrete Specimens.

[28] (2016). Standard Test Method for Pulse Velocity Through Concrete.

[29] Ali F., Khan M.A., Qurashi M.A., Shah S.A., Khan N.M., Khursheed Z., Rahim H.S., Arshad H., Farhan M., Waseem M. (2020). Utilization of Pyrolytic Carbon Black Waste for the development of Sustainable Materials. Processe.

[30] Hwang E., Kim G., Choe G., Yoon M., Gucunski N., Nam J. (2018). Evaluation of concrete degradation depending on heating conditions by ultrasonic pulse velocity. Constr. Build. Mater..

[31] Ali Z., Bhaskar S.B. (2016). Basic statistical tools in research and data analysis. Indian J. Anaesth..

[32] Voigt T., Malonn T., Shah S.P. (2006). Green and early age compressive strength of extruded cement mortar monitored with compression tests and ultrasonic techniques. Cem. Concr. Res..

[33] Bentz P. (2009). Influence of internal curing using lightweight aggregates on interfacial transition zone percolation and chloride ingress in mortars. Cem. Concr. Compos..

[34] Kumar S., Kapoor K., Singh S.P., Singh P., Sharma V. (2022). A review on the properties of natural and recycled coarse aggregates concrete made with different coal ashes. Clean. Mater..

[35] Vargas P., Baena O.R., Tobón J.I. (2017). Microstructural analysis of interfacial transition zone (ITZ) and its impact on the compressive strength of lightweight concretes. Constr. Build. Mater..

[36] Gündüz L. (2008). The effects of pumice aggregate/cement ratios on the low-strength concrete properties. Constr. Build. Mater..

[37] Newman J., Owens P. (2003). Properties of lightweight concrete. Adv. Concr. Technol..

[38] Lo T.Y., Cui H.Z., Tang W.C., Leung W.M. (2008). The effect of aggregate absorption on pore area at interfacial zone of lightweight concrete. Constr. Build. Mater..

[39] Alonso C., Femandez L. (2004). Dehydration and rehydration processes of cement paste exposed to high temperature environments. J. Mater. Sci..

[40] Kim G.Y., Kang Y.W., Lee T.G., Choe G.C., Yoon M.H. (2012). An Experimental Study on the Mechanical Properties of Concrete with High Temperatures and Cooling Conditions. J. Korea Inst. Build. Constr..

[41] Roufael G., Beaucour A., Eslami J., Hoxha D., Noumowé A. (2021). Influence of lightweight aggregates on the physical and mechanical residual properties of concrete subjected to high temperatures. Constr. Build. Mater..

[42] Zhang J., Shen Y., Yang G., Zhang G., Wang Y., Hou X., Sun Q., Li G. (2021). Inconsistency of changes in uniaxial compressive strength and P-wave velocity of sandstone after temperature treatments. J. Rock Mech. Geotech. Eng..

[43] Hossain K.M.A. (2004). Properties of volcanic pumice based cement and lightweight concrete. Cem. Concr. Res..

[44] Torić N., Boko I., Juradin S., Baloević G. (2016). Mechanical properties of lightweight concrete after fire exposure. Struct. Concr..

[45] Rao S.K., Sravana P., Rao T.C. (2016). Experimental studies in Ultrasonic Pulse Velocity of roller compacted concrete pavement containing fly ash and M-sand. Int. J. Pavement Res. Technol..

[46] Lee T., Lee J., Choi H. (2020). Assessment of Strength Development at Hardened Stage on High-Strength Concrete Using NDT. Appl. Sci..

[47] Abed M., Brito J. (2020). Evaluation of high-performance self-compacting concrete using alternative materials and exposed to elevated temperatures by non-destructive testing. J. Build. Eng..

